# The effects of flattening filter‐free beams and aperture shape controller on the complexity of conventional large‐field treatment plans

**DOI:** 10.1002/acm2.14108

**Published:** 2023-08-01

**Authors:** Cheukkai B. Hui, Amir Pourmoghaddas, Yildirim D. Mutaf

**Affiliations:** ^1^ Department of Radiation Oncology Kaiser Permanente Dublin California USA

**Keywords:** ASC, complexity, FFF beams, planning study

## Abstract

**Purpose:**

The purpose of this study was to investigate the impact of using flattening filter‐free (FFF) beams and the aperture shape controller (ASC) on the complexity of conventional large‐field treatment plans.

**Methods and materials:**

A total of 24 head and neck (H&N) and 24 prostate with pelvic nodes treatment plans were used in this study. Each plan was reoptimized using the original clinical objectives with both flattened and FFF beams, as well as six different ASC settings. The dosimetric qualities of each plan cohort were evaluated using commonly used dose‐volume histogram values, and plan complexities were assessed through metrics including monitor unit (MU)/Dose, change in gantry speed, multileaf collimator (MLC) speed, the edge area ratio metric (EM), and the equivalent square length.

**Results:**

No significant differences in dosimetric qualities were found between plans with flattened and FFF beams. The ASC settings did not have significant effects on dosimetric qualities in the H&N plan cohort, but the “very high” ASC setting resulted in poorer dosimetric results for the prostate plans. Plans with FFF beams had significantly higher MU/Dose compared to plans with flattened beams. The use of flattening filter (FF) had significant effects on the change in gantry speed, with flattened beams producing plans that required higher change in gantry speed. However, the FF did not have significant effects on MLC speed, EM, or equivalent square length. In contrast, ASC settings had significant effects on these three metrics; increasing the ASC level resulted in plans with decreasing MLC speed, lower edge area ratio, and higher equivalent square length.

**Conclusion:**

This study demonstrated that using FFF beams with various ASC settings, except for the “very high” level, can produce plans with reduced complexities without compromising dosimetric qualities in conventional large‐field treatment plans.

## 1 INTRODUCTION

A standard medical linear accelerator generates a uniform intensity beam by placing a flattening filter (FF) to facilitate treatment planning. However, with advances in optimization algorithms, intensity modulated radiation therapy (IMRT) plans can now be generated by modulating dose rates and multileaf collimator (MLC) patterns. This allows for conformal dose distribution using flattening filter‐free (FFF) beams with IMRT techniques.

FFF beams have a cone‐shaped profile and a higher maximum dose rate, which may speed up treatment time for high dose and highly modulated treatment plans. This faster treatment time may reduce the impact of patient and anatomical motion during treatment.^1^ A higher dose rate may potentially improve volumetric modulated arc therapy (VMAT) optimization by providing a wider range of parameters for plan optimization.^2^ In addition to faster treatment time and improved optimization, FFF beams have a sharper penumbra compared to flattened beams,^3^, ^4^ which can result in lower peripheral dose near the edges of treatment fields.^3^, ^5^, ^6^ At distances further away from the treatment field, out‐of‐field dose is largely reduced in FFF mode due to substantial reduction in head leakage.^5^, ^6^, ^7^ Overall, FFF beams contribute lower out‐of‐field dose to patients and can potentially reduce the long‐term risk of secondary malignancies.

Early implementations of FFF beams were primarily used in high dose treatments such as stereotactic radiosurgery (SRS) and stereotactic body radiation therapy (SBRT) to achieve faster treatment times.^8^, ^9^, ^10^, ^11^, ^12^ The small field sizes in SRS and SBRT allow for conformal target coverage with minimally modulated FFF beams. Currently, FFF beam treatment plans have been implemented in various disease sites and treatment techniques. Many studies have concluded that the dosimetric qualities are similar using either FFF or flattened beams in treatment plans of different sites and treatment techniques.^13^, ^14^, ^15^, ^16^, ^17^, ^18^, ^19^

Treatment plans with FFF beams generally require more monitor unit (MU) due to their heterogenous intensity profile.^13^, ^15^, ^16^, ^17^, ^18^ Plans with higher MUs have been associated with increased field modulation and aperture complexity.^20^ Several studies have reported that FFF beam plans require more aperture segments than flattened beam plans,^13^, ^18^ implying a higher degree of complexity. Plan complexity is generally defined by machine parameters (MLC, gantry, and dose rate variation) and properties of the MLC leaf apertures. Highly complex fields are generally created by rapid changes in machine parameters. However, large variations in machine parameters could lead to reduced accuracy in treatment delivery.^21^, ^22^, ^23^, ^24^ Conversely, highly complex apertures may also reduce the accuracy of dose calculation by the treatment planning system (TPS),^25^ because the uncertainty in MLC modeling can be magnified when confined to a small or irregular field aperture. Some studies have also reported that high complexity plans could be less robust to motion and more susceptible to interplay effects.^26^, ^27^

To reduce plan complexity, the aperture shape controller (ASC) was recently introduced in the Eclipse TPS (Varian Medical Systems, Palo Alto, CA). It is a component in the leaf sequencer and is available in VMAT optimization using the photon optimizer (PO) algorithm v15.5 or newer. The ASC controls field complexity by penalizing large local curvature formed by the tips of adjacent MLC leaves. The penalty is incurred into the cost function of the optimization. The magnitude of the penalty is controlled by the ASC level, with six settings from “off” (default) to “very high”. Studies have shown that ASC could reduce treatment plan complexity with minimal compromise on plan quality.^28^, ^29^, ^30^

The purpose of this study is to investigate the impacts of the FF and ASC settings on conventional treatment plans with large field sizes. Specifically, we aim to demonstrate that the use of FFF beams with the help of ASC can produce treatment plans with similar dosimetric qualities and plan complexities, allowing conventional treatment plans to benefit from FFF beam delivery without a potential penalty in plan complexity. The study consists of two parts: first, examining the effects of the FF and ASC settings on dosimetric qualities; and second, evaluating their effects on plan complexity.

## 2 METHODS AND MATERIALS

### 2.1 Plan optimization

For this study, 24 head and neck (H&N) and 24 prostate with pelvic nodes VMAT treatment plans were sequentially selected from our clinical data base. The selection criteria required that H&N plans have three prescription dose levels, while prostate plans have two prescription levels for the prostate and pelvic nodes. Tables 1 and 2 summarize the properties of the selected clinical plans. All plans were originally planned for Truebeam machines (Varian Medical Systems) and optimized with Eclipse TPS. Optimization was performed using the PO algorithm (version 16.1.0) with both ASC and convergence mode turned off. The optimization restarted at MR3. The maximum gantry speed was 6°/s, while the maximum dose rate was 600 MU/min for the flattened beam plans. Jaw tracking was enabled for all plans.

**TABLE 1:** Summary of the clinical plan parameters in the head and neck cohort.

H&N plans (*N* = 24)	
**PTV volumes**	**μ ± σ (cc)**
1st PTV	111.9 ± 136.9
2nd PTV	393.7 ± 259.9
3rd PTV	274.3 ± 102.3
PTV_tot_	647.8 ± 281.9
**Prescription**	**(# of plans)**
7000/6300/5600 cGy in 35fx	24
**Plan properties**	
Beam energy	6 MV flattened
MLC type	Varian HD 120
MLC widths	in: 32 cm × 0.25 cm out: 28 cm × 0.5 cm
**Number of arcs**	**(# of plans)**
3 arcs	17
4 arcs	7
**Arc 1 and 2 collimator angle**	**(# of plans)**
5°, −5°	3
8°, −8°	1
10°, −10°	10
15°, −15°	4
20°, −20°	3
30°, −30°	2
15°, −20°	1
**Arc 3 and 4 collimator angle**	**(# of plans)**
90°	23
85°	1

Abbreviations: H&N, head and neck; MLC, multileaf collimator; PTV, planned target volume.

**TABLE 2:** Summary of the clinical plan parameters in the prostate cohort.

Prostate plans (*N* = 24)	
**PTV volumes**	**μ ± σ (cc)**
1st PTV	130.7 ± 56.1
2nd PTV	973.3 ± 237.3
PTV_tot_	1073.8 ± 212.8
**Prescription**	**(# of plans)**
7000/5040 cGy in 28 fx	20
6000/4400 cGy in 20 fx	4
**Plan Properties**	
Beam Type	10 MV flattened
MLC type	Millennium 120
MLC widths	in: 40 cm × 0.5 cm out: 20 cm × 1.0 cm
**Number of arcs**	**(# of plans)**
2 arcs	5
3 arcs	14
4 arcs	5
**Arc 1 and 2 collimator angle**	**(# of plans)**
10°, −10°	7
15°, −15°	4
20°, −20°	8
25°, −25°	2
30°, −30°	3
**Arc 3 and 4 collimator angle**	**(# of plans)**
90°	18
75°, −75°	1

Abbreviations: MLC, multileaf collimator; PTV, planned target volume.

To prepare for plan optimization for the study, the clinical beam configurations of the selected plans were retained, except that their beam control points were cleared. Due to the use of jaw tracking during clinical plan optimization, the clinical field sizes may have been smaller than necessary for optimal planning. As such, field sizes were increased in each cleared plan to encompass a larger portion of the planned target volume (PTV). The maximum x‐field size of all beams was set to 15 cm; while the maximum y‐field size was adjusted to encompass the PTV along the collimator y‐dimension. In the H&N plan cohort, the resulting maximum y‐field size ranged from 16 to 22 cm, while in the prostate plan cohort it ranged from 18.5 to 30 cm. Each cleared plan was then duplicated into 12 different versions: half were optimized with flattened beams and half with FFF beams (1400 MU/min for 6 MV FFF beams and 2400 MU/min for 10 MV FFF beams). Six different ASC settings (off—0, very low—1, low—2, moderate—3, high—4, very high—5) were applied to the two beam types. The plans were optimized with the original clinical optimization objectives. To ensure that the target coverage converged to the optimal level, each plan underwent four consecutive optimizations, with the dose from the previous round used as an intermediate dose in the subsequent round. Dose calculation was performed using the Anisotropic Analytical Algorithm (version 16.1.0). Finally, all plans were normalized to 100% prescribed dose covering 95% of the primary PTV.

### 2.2 Plan quality

The dosimetric qualities were evaluated by comparing commonly used dose‐volume histogram (DVH) values. For the H&N plans, the following doses and dose volumes were assessed:

1. D_95%_ of the secondary and tertiary PTVs,
2. Conformity index = Isodose volume of tertiary prescription /combined PTV volume,
3. Isodose volume of 50% of tertiary prescription,
4. D_0.03 cc_ of the spinal cord,
5. D_0.03 cc_ of the brainstem,
6. D_0.03 cc_ of the left and right brachial plexuses,
7. D_mean_ of the left and right parotids,
8. D_0.03 cc_ of the mandible,
9. D_0.03 cc_ of the lips,
10. D_mean_ of the oral cavity,
11. D_mean_ of the larynx,
12. D_mean_ of the pharynx constrictor.

For the prostate plans, the following doses and dose volumes were assessed:

1. D_95%_ of the secondary PTV,
2. Conformity index = Isodose volume of secondary prescription /combined PTV volume,
3. Isodose volume of 50% of secondary prescription,
4. V_4000_, V_5000_, and V_6000_ of the bladder,
5. V_4000_, V_5000_, and V_6000_ of the rectum,
6. V_4000_ and D_0.03 cc_ of the bowel space,
7. D_5%_ of the left and right femurs,
8. D_0.03 cc_ of the penile bulb.

### 2.3 Plan complexity

Without a clear consensus, many complexity metrics have been proposed in recent years to quantify the complexity of an IMRT plan.^31^ These complexity metrics can generally be divided into three categories based on their approaches: fluence, deliverability, and accuracy.^32^ Fluence metrics quantify complexity by evaluating the heterogeneity of the fluence. However, they can be ineffective since a homogeneous fluence could be generated by combinations of complex fields. Deliverability metrics evaluate the mechanical and dosimetric parameters of the machine such as change in gantry speed, MLC speed and MU. Accuracy metrics, also known as aperture complexity metrics, focus on open MLC leaf apertures and identify fields with small and irregular apertures, as the accuracy of dose calculations for such fields decreases.

Although aperture complexities from different literature sources take on different forms, many of them quantify open apertures in a similar fashion. Originally introduced as a penalization term in optimization, the edge area ratio metric (EM) was proposed by Younge et al to quantify field complexity.^33^ The EM is defined as:

(1) $$EM\;=\frac{1}{\sum_{i}MU_{i}}\;\sum_{i}MU_{i}\frac{y_{i}}{A_{i}}$$

where *i* is the index of the control point; $MU_{i}$ is the number of MU; $y_{i}$ is the perimeter of the open apertures excluding leaf ends; and $A_{i}$ is the area of the open apertures. Equation (1) is weighted by MU and its components are intuitive: increasing irregularity (increasing $y_{i}$) and lowering aperture size (decreasing $A_{i}$) will contribute to higher EM. In addition, Studies have shown correlation between EM and gamma passing rate.^34^, ^35^ Therefore, the EM value would be evaluated in our analysis.

**FIGURE 1:**
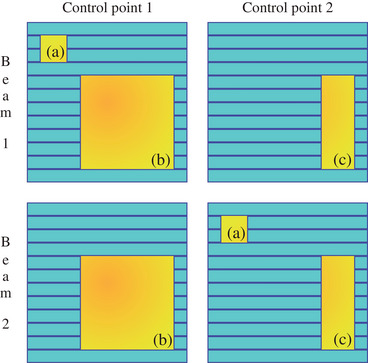
MLC segments of two different beams, each composed of only two control points. Both beams have three identical apertures: A (2 mm MLC length, 1 mm^2^ area), B (20 mm MLC length, 100 mm^2^ area), and C (4 mm MLC length, 20 mm^2^ area). Aperture A is located on the first control point of beam 1 and the second control point of beam 2. Both beams have identical MU per control point. In principle, the aperture complexity metrics of these two beams should be the same, but their traditional EMs differ. The proposed equivalent square length neutralizes the aperture distribution effects and yields the same value for both beams. EM, edge area ratio metric; MLC, multileaf collimator.

One limitation of Equation (1) is that its value can be affected by the distribution of open apertures across control points. For example, consider the scenario depicted in Figure 1. Each of these two beams consists of only two control points and has the same three apertures, with each control point contributing the same MU. In principle, both beams should have the same aperture complexity. However, when using Equation (1), their EM values are different due to the different distributions of apertures. To address this issue, we modified the EM formula to allow for equal evaluation of each aperture, regardless of its distribution among control points:

(2) $$EM_{mod}=\frac{\sum_{i,j}MU_{i}\cdot y_{ij}}{\sum_{i,j}MU_{i}\cdot A_{ij}}\;$$

where *j* is the index of each open aperture within control point *i*. Furthermore, we rearranged Equation (2) to present it as equivalent square length (ESQL):

(3) $$ESQL\;=\frac{2}{EM_{mod}}\;=\frac{2\sum_{i,j}MU_{i}\cdot A_{ij}}{\sum_{i,j}MU_{i}\cdot y_{ij}}\;$$

If a plan has its ESQL equal to 5 mm, it implies that its aperture complexity is equivalent to a plan delivered by a 5 mm^2^ square field. The EQSL is preferred over EM_mod_ because it provides a more intuitive representation.

In addition to the EM and ESQL values, three other metrics were selected to quantify plan complexities in this study. In summary, the following five complexity metrics were evaluated on the plans:

1. the MU to prescribed dose ratio (MU/Dose),
2. the MU‐weighted average change in gantry speed,
3. the MU‐weighted average MLC leaf speed,
4. the EM,
5. and the ESQL.

### 2.4 Statistical analyses

To evaluate the effects of both FF and ASC settings, a two‐way analysis of variance (ANOVA) was employed. This statistical test enables the examination of the impact of two independent variables (FF and ASC) on the dependent variable (e.g., MU/Dose) and any interaction effect between them. The interaction term in a two‐way ANOVA indicates whether the influence of one independent variable on the dependent variable is consistent across all values of the other independent variable. A statistically significant interaction suggests that the relationship between one independent variable and the dependent variable is contingent on the value of the other independent variable. In contrast, if the interaction effect is statistically insignificant, the main effects of each independent variable can be interpreted independently. If the main effects are statistically significant, it implies that each independent variable has a significant impact on the dependent variable, irrespective of the value of the other independent variable. In cases where a statistically significant interaction is observed, post‐hoc analyses can be conducted to determine the source of differences between the independent variables. If the interaction effect is not significant but the main effects are, post‐hoc analyses can be used to compare the means of each level of the independent variables separately. In this study, a repeated measures ANOVA test was utilized as all comparisons were paired, and a significance level of 0.05 was set for all comparisons.

To test the hypothesis that neither FF nor ASC settings affected DVH values, a repeated measures two‐way multivariate ANOVA (MANOVA) with Wilks’ lambda was employed. MANOVA differs from ANOVA in that it allows for the simultaneous analysis of multiple dependent variables to determine the effects of independent variables. In the event that the hypothesis was rejected, post‐hoc analyses would be conducted to identify affected DVH values and determine the source of any observed differences.

For complexity metrics, a repeated measures two‐way ANOVA was performed. Given the expectation of significant differences in some complexity metrics between different ASC settings, ANOVA tests were conducted directly rather than starting with MANOVA. If statistically significant differences were observed, post‐hoc analyses would be conducted to determine their effects on complexity metrics.

## 3 RESULTS

### 3.1 Plan quality

Table 3 presents the results of the MANOVA test examining the effects of FF and ASC settings on dosimetric qualities. The analysis revealed no statistically significant interaction effects between these two factors. However, while the main effects analysis indicated that the FF had no statistically significant impacts on dosimetric qualities, ASC settings were found to have statistically significant effects in both treatment site cohorts.

**TABLE 3:** Summary of multivariate analysis of variance results on effects of flattening filter and aperture shape controller settings on dosimetric qualities.

	*p*‐value	
Effect	H&N plans	Prostate plans
Flattening filter	0.087	0.307
ASC settings	<0.001^*^	<0.001^*^
Interactions	0.056	0.123

Abbreviations: ASC, aperture shape controller; H&N, head and neck.

* Indicates significant difference (*p* < 0.05).

Further details of the post‐hoc analyses can be found in Appendix A. In the H&N plan cohort, post‐hoc analyses suggested that although DVH values varied between plans with different ASC settings, these differences were likely due to stochastic effects rather than ASC effects. In contrast, post‐hoc ANOVA tests for the prostate plan cohort indicated that ASC settings had significant impacts on 12 out of 14 DVH values. Plans optimized with ASC‐5 exhibited the poorest dosimetric qualities in terms of low dose region and dose to organs at risk (OAR)s, with pairwise *t*‐tests revealing significant differences in DVH values compared to plans with other ASC settings. Plans optimized with ASC‐4 had the second poorest dosimetric qualities for most DVH values, but *t*‐tests showed mostly insignificant differences compared to plans with ASC‐0 to 3 settings.

### 3.2 Plan complexity

Tables 4 and 5 summarize the *p*‐values of individual ANOVA test results on complexity metrics in the H&N and prostate plan cohorts, respectively. The corresponding post‐hoc analysis results are provided in Appendix B.

**TABLE 4:** Summary of analysis of variance results on plan complexities in the head and neck cohort.

	*p*‐value				
Effect	MU/Dose	Δv_G_ ^a^	v_MLC_	EM	ESQL
Flattening filter	<0.001^*^	<0.001^*^	0.198	0.977	0.460
ASC settings	<0.001^*^	<0.001^*^	<0.001^*^	<0.001^*^	<0.001^*^
Interactions	0.159	<0.001^*^	0.001^*^	0.019^*^	0.730

Abbreviations: ASC, aperture shape controller; EM, edge area ratio metric; EQSL, equivalent square length; MLC, multileaf collimator.

* Indicates significant difference (*p* < 0.05).

^a^ change in gantry speed.

**TABLE 5:** Summary of analysis of variance results on plan complexities in the prostate cohort.

	*p*‐value				
Effect	MU/Dose	Δv_G_ ^a^	v_MLC_	EM	ESQL
Flattening filter	<0.001^*^	<0.001^*^	0.418	0.181	0.163
ASC settings	0.077	<0.001^*^	<0.001^*^	<0.001^*^	<0.001^*^
Interactions	0.959	<0.001^*^	<0.001^*^	0.002^*^	0.220

Abbreviations: ASC, aperture shape controller; EM, edge area ratio metric; EQSL, equivalent square length; MLC, multileaf collimator.

* Indicates significant difference (*p* < 0.05).

^a^ Change in gantry speed.

The FF had a statistically significant effect on the traditional MU/Dose ratio. On average, the MU/Dose was 4.6 ± 1.1 for FFF beam plans and 3.9 ± 0.8 for flattened beam plans in the H&N plan cohort; and 4.8 ± 1.2 for FFF beam plans and 3.5 ± 0.7 for flattened beam plans in the prostate plan cohort. Among the H&N plans, different ASC settings also yielded small but significant differences in MU/Dose ratio, with ASC‐2 yielding the minimum MU/Dose at 4.2 ± 1.1 and ASC‐5 yielding the highest MU/Dose at 4.4 ± 1.1.

**FIGURE 2:**
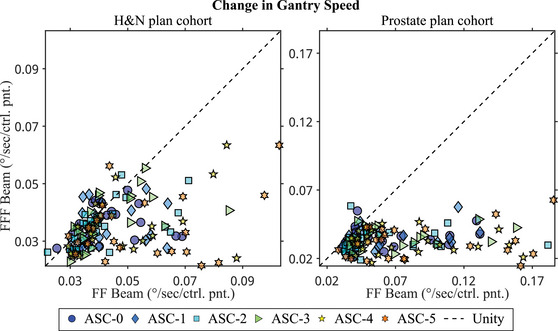
Scatter plots of change in gantry speed for the H&N plan cohort (left) and the prostate plan cohort (right). The horizontal and vertical axes represent different flattening filter groups, while different marker types represent different ASC settings. Most data points fall below the unity line, indicating that changes in gantry speed were higher for FF beam plans compared to FFF beam plans. Additionally, differences between FF and FFF beams were more pronounced in the prostate plan cohort. FF, flattening filter; FFF, flattening filter‐free; H&N, head and neck.

There were statistically significant interaction effects between the FF and ASC settings on gantry speed change in both treatment site cohorts. Figure 2 shows the scatter plots of gantry speed change. As shown in the figure and post‐hoc analyses, FFF beams produced plans requiring significantly lower gantry speed change. On average, plans with FFF beams reduced gantry speed change by 21% in the H&N plan cohort and 51% in the prostate plan cohort. Among FFF beam plans, different ASC settings did not result in different gantry speed changes. However, among flattened beam plans, the ASC‐5 setting produced plans requiring significantly higher gantry speed change.

**FIGURE 3:**
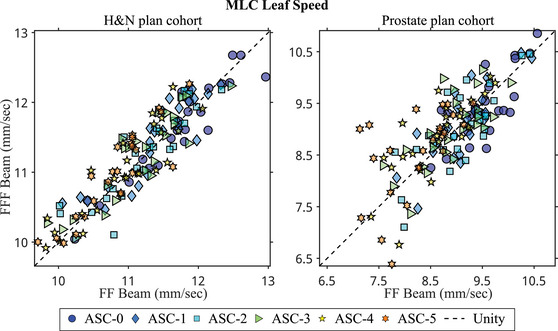
Scatter plots of MLC leaf speed for the H&N plan cohort (left) and the prostate plan cohort (right). The horizontal and vertical axes represent different flattening filter groups, while different marker types represent different ASC settings. Most data points fall along the unity line, indicating that the flattening filter did not affect MLC leaf speed. In both treatment site cohorts, there was a trend of decreasing MLC leaf speed with increasing ASC levels. ASC, aperture shape controller; MLC, multileaf collimator; H&N, head and neck.

There were statistically significant interaction effects on MLC leaf speed. Figure 3 displays scatter plots of MLC leaf speed. In general, H&N plans required faster MLC leaf movements than prostate plans. As shown in the figure and post‐hoc analyses, MLC leaf speed gradually reduced with increasing ASC levels, with most reductions being significant. Comparing to ASC‐0, applying the ASC‐5 setting reduced the MLC leaf speed by an average of 7% in the H&N plan cohort and 12% in the prostate plan cohort. Among H&N plans optimized with ASC‐5, FFF beam plans required significantly higher MLC leaf speed than flattened beam plans. A similar trend was observed in the prostate plan cohort, although the difference was not significant.

**FIGURE 4:**
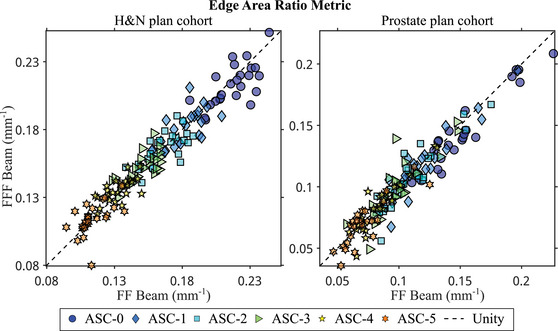
Scatter plots of the EM for the H&N plan cohort (left) and the prostate plan cohort (right). The horizontal and vertical axes represent different flattening filter groups, while different marker types represent different ASC settings. Most data points fall along the unity line, indicating that the flattening filter did not affect EM. In both treatment site cohorts, there was a clear trend of decreasing EM values with increasing ASC levels. In the prostate plan cohort, data points for ASC‐0 fell below the unity line, suggesting that FF beam plans might yield higher EM values than FFF beam plans when using the ASC‐0 setting. ASC, aperture shape controller; EM, edge area ratio metric; FF, flattening filter; FFF, flattening filter‐free; H&N, head and neck.

**FIGURE 5:**
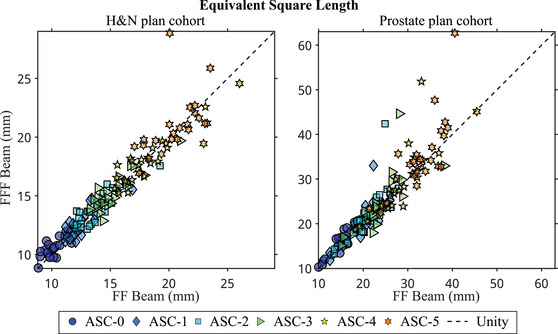
Scatter plots of the EQSL for the H&N plan cohort (left) and the prostate plan cohort (right). The horizontal and vertical axes represent different flattening filter groups, while different marker types represent different ASC settings. Most data points fall along the unity line, indicating that the flattening filter did not affect EQSL. In both treatment site cohorts, there was a clear trend of increasing EQSL values with increasing ASC levels. ASC, aperture shape controller; EQSL, equivalent square length; H&N, head and neck.

Figures 4 and 5 show scatter plots of EM and ESQL, respectively. Overall, H&N plans required smaller and more complex apertures than prostate plans. FF had no statistically significant effect on neither EM nor ESQL. However, ASC settings had statistically significant effects on both aperture complexity metrics, with increasing ASC levels resulting in decreasing EM and increasing EQSL. Among H&N plans, applying ASC increased EQSL value from 10.4 mm (ASC‐0) to 12.6 mm (ASC‐1), 15.3 mm (ASC‐3), and 20.9 mm (ASC‐5). Similarly, among prostate plans, EQSL value increased from 15.3 (ASC‐0) to 19.7 mm (ASC‐1), 24.2 mm (ASC‐3), and 34.6 mm (ASC‐5). There were statistically significant interaction effects on EM. Among prostate plans optimized with ASC‐0, FFF beam plans resulted in significantly lower EM than flattened beam plans. No such trend was observed in the H&N plan cohort. There were no statistically significant interaction effects on ESQL.

## 4 DISCUSSION

In this study, we investigated the combined effects of FF and ASC settings on the quality and complexity of conventional large‐field treatment plans. To our knowledge, this is the first study to examine their combined effects. To ensure clinical relevance and unbiased results, treatment plans were optimized with their original clinical objectives. A relatively large number of treatment plans were processed and paired tests were used for comparisons to increase statistical power.

The use of FFF beams for large‐field treatment plans has been a topic of concern due to the potential dosimetric differences compared to flattened beams. However, previous studies have shown that FFF beams can generate H&N and prostate plans with equivalent target dose coverage.^13^, ^14^, ^15^, ^16^, ^17^, ^18^ These studies also concluded that other plan qualities and doses to OARs were mostly similar, although significant differences in some tested DVH values were reported. The reported differences varied between studies, which may be attributed to the small study sizes and lack of multiple comparison correction. In our study, we used MANOVA on a relatively large sample size and found that the FF had no significant effects on the dosimetric qualities of conventional large‐field treatment plans. The results confirmed that FFF beams can be used to generate high‐quality large‐field treatment plans.

It has been assumed that applying ASC in optimization could compromise dosimetric qualities by prohibiting the MLC leaves from forming irregular apertures. However, Scaggion et al. showed that plan quality was not degraded by using the “very high” ASC setting on prostate and H&N plans^30^; although their prostate plans did not include pelvic nodes, so their results could not be applied to plans of larger field sizes. Binny et al. also showed that using different ASC settings could produce H&N and pelvis plans with similar dosimetric qualities,^28^ albeit with a limited number of plans and no statistical comparison. In our study, we showed that dosimetric qualities were not affected by ASC settings in the H&N plan cohort, and there were no significant differences in dosimetric qualities using ASC settings up to the “high” level in the prostate plan cohort. However, using the “very high” ASC setting deteriorated the dosimetric qualities of prostate plans but not H&N plans. This discrepancy may be caused by differences in target size, photon energy, and the MLC leaf width between the two plan cohorts.

The MU/Dose ratio was significantly higher for FFF beam plans than for flattened beam plans. This increase was more prominent in the prostate plan cohort, which reaffirms that larger targets might require more MU to compensate for the lower beam intensity at the peripheries of the FFF fields.

There were no statistically significant differences in MLC leaf speed between FFF beam plans and flattened beam plans. However, higher ASC settings reduced the demand for MLC leaf speed, which could potentially reduce MLC position errors during treatment delivery.^21^, ^22^, ^23^, ^24^ We also found that plans with FFF beams required smaller changes in gantry speed compared to those with flattened beams. This could be due to the lower dose rate of flattened beams, which required the gantry to slow down for high‐MU segments. Reducing the changes in gantry speed could potentially improve treatment accuracy, as rapid acceleration and deceleration of the gantry may cause discrepancies between the planned and delivered gantry angles.^21^, ^23^ Interestingly, the ASC settings had a significant effect on the changes in gantry speed only for the flattened beam plans. Specifically, the “very high” ASC setting resulted in a higher change in gantry speed for the flattened beam plan, but not for the FFF beam plan.

Concerns regarding higher aperture complexity associated with FFF beams have been raised, especially for large‐field treatment plans. However, our study showed that FFF beams did not require more complicated apertures than flattened beams. On the other hand, we found that increasing the ASC level produced plans with decreasing aperture complexity. This is consistent with the findings of previous studies.^28^, ^29^, ^30^

One limitation of using the original EM as a metric to evaluate aperture complexity was that it could diminish the weighting of small and irregular apertures if they were in the same control point as a large aperture. We proposed the modified EM described in Equation (2) to remove this distribution dependency. Therefore, unless small and irregular apertures were disproportionately distributed across control points, the modified EM would be very close to the original EM. Furthermore, since the proposed EQSL was the inverse of the modified EM, the scatter plots in Figures 4 and 5 appear to be inverse of each other. We proposed using EQSL because it allows us to interpret aperture complexity as a simple field size. This makes it easier to adjust our expectations of EQSL based on target size.

It is important to point out that the results of this study were only applicable to VMAT treatment plans optimized using the PO algorithm in the Eclipse TPS. Different optimization algorithms could produce different results. For example, some optimization algorithms might generate less complex treatment plans than those produced using the Eclipse ASC‐5 setting. If plan complexity became low enough, FFF beam plans might require higher aperture complexity than flattened beam plans to achieve similar dosimetric qualities. However, since dosimetric qualities were already poorer among prostate plans using the “very high” ASC setting, they would likely degrade further in plans with even lower plan complexities.

This study followed the RATING guideline and had a score of 89%.^36^

## 5 CONCLUSION

In conclusion, our study showed that plans with FFF beams had equal dosimetric qualities and aperture complexity compared to plans with flattened beams. FFF beam plans also had lower change in gantry speed owing to their higher dose rate. Therefore, using FFF beams in large‐field treatment planning should be encouraged without the concern of compromising dosimetric qualities or plan complexities. This is especially true when properties of FFF beams are beneficial for treatment delivery. Additionally, our study showed that applying the ASC setting up to the “high” level could reduce plan complexity while maintaining the same dosimetric qualities.

## AUTHOR CONTRIBUTIONS

The study was designed by Cheukkai Hui. Data processing was conducted by all three authors, and data analysis was performed by Cheukkai Hui and Amir Pourmoghaddas. The manuscript was drafted by Cheukkai Hui.

## CONFLICT OF INTEREST STATEMENT

The authors have no conflict of interest.

## A Post‐hoc Analyses in Plan Quality

To identify which specific DVH values were affected by ASC settings, we performed one‐way ANOVA tests with Benjamini‐Hochberg (B–H) correction. In cases where we identified a significant effect of ASC settings on a particular DVH value, we conducted additional pairwise *t*‐tests with B–H correction to further investigate the relationship between ASC settings and that DVH value.

Table A1 summarizes the adjusted *p*‐values for the one‐way ANOVA tests conducted on the H&N plan cohort. Significant differences were observed in the D_95%_ of the tertiary PTV, conformity index and D_0.03cc_ of the right brachial plexus. The results of pairwise *t*‐tests performed on these three DVH values are summarized in Tables A2, A3, A4. Table A2 indicates that a significant difference in D_95%_ was only present between plans utilizing ASC‐4 and ASC‐5 settings, with no significant differences observed in other comparisons. Similarly, Table A4 reveals a single significant difference in D_0.03cc_ between plans employing ASC‐0 and ASC‐4 settings. No significant differences were detected in conformity index. This suggests that the observed significant differences in DVH values may be attributed to randomness.

Table A5 summarizes the adjusted *p*‐values for one‐way ANOVA tests conducted on the prostate plan cohort. Results indicate that ASC settings significantly impacted all DVH values, with the exception of D_95%_ for the secondary PTV and D_0.03cc_ for the penile bulb. Tables A6, A7, A8, A9, A10, A11, A12, A13, A14, A15, A16, A17 provide summaries and *t*‐test *p*‐values for the 12 DVH values that exhibited significant differences. The data reveals that plans generated using the ASC‐5 setting had the highest conformity index, largest low dose volumes, and highest dose to OARs, with most differences being statistically significant. The ASC‐4 setting generated plans with the second‐worst values for 8 of the 12 tested DVH values; however, these differences were mostly not statistically significant when compared to plans using ASC‐0 to 3 settings.

## B Post‐hoc Analyses in Plan Complexity

The post‐hoc analyses were used to identify specific effects that resulted in different plan complexities, and they varied slightly depending on the initial ANOVA results. If the flattening filter and ASC settings had statistically significant interaction effects on plan complexity, we performed pairwise *t*‐tests with B‐H correction to compare each possible combination of flattening filter and ASC settings. If there were no statistically significant interaction effects, we performed pairwise *t*‐tests only on the main effects that had statistically significant effects in the ANOVA test. However, in our comparison, we didn't need to perform additional main effect analyses for the flattening filter effects because there were only two options. For comparisons involving ASC settings, we only showed test results between adjacent ASC settings because we wanted to focus on the effects of increasing ASC settings. We still applied *t*‐tests and B‐H corrections to all comparisons.

Plans with FFF beams had higher MU/Dose compared to plans with flattened beams, and the differences were statistically significant. Table 4 shows that ASC settings also had a significant effect on MU/Dose in the H&N plan cohort, but not in the prostate plan cohort as shown in Table 5. Table B1 shows a summary and *t*‐test *p*‐values between plans with adjacent ASC settings. As shown in Table B1, MU/Dose appeared to decrease from ASC‐0 to 2 and increase from ASC‐2 to 4.

Tables 4 and 5 show that there were statistically significant interaction effects between flattening filter and ASC settings on changes in gantry speed. Tables B2 and B3 show *t*‐test *p*‐values between plans with adjacent ASC settings for both flattened and FFF beams, as well as *t*‐test *p*‐values between plans with flattened and FFF beams for each ASC setting. We found significant differences in gantry speed change between plans with flattened and FFF beams for all ASC settings in both treatment site cohorts. In the H&N plan cohort, we found significant differences between plans with ASC‐4 versus 5 settings, but only among plans with flattened beams. Similarly, in the prostate plan cohort, we found significant differences between plans with ASC‐3 versus 4 and 4 versus 5 settings, but only among plans with flattened beams.

Tables B4 and B5 show *t*‐test *p*‐values for post‐hoc comparisons of MLC leaf speed. We found that different ASC settings resulted in significantly different MLC leaf speeds in most cases for both treatment site cohorts. Among H&N plans, we also found significant differences between flattened and FFF beam plans with the ASC‐5 setting. However, we found no significant differences between flattened and FFF beam plans in the prostate plan cohort.

Tables B6 and B7 show *t*‐test *p*‐values for post‐hoc comparisons of EM. In both treatment site cohorts, we found that different ASC settings resulted in significantly different EM values in all comparisons. In the prostate plan cohort, we found significant differences between flattened and FFF beam plans with the ASC‐0 setting. However, we found no significant differences between flattened and FFF beam plans with any ASC setting in the H&N plan cohort.

We performed main effect analyses on the effects of ASC settings on EQSL and presented the results in Tables B8 and B9. These tables show that different ASC settings resulted in significantly different EQSL values in all comparisons.

**TABLE A1:** Summary of post‐hoc analysis of variance results on effects of aperture shape controller settings on head and neck plan dose‐volume histogram values.

DVH value	Adjusted *p*‐value
2nd PTV D_95%_	0.260
3rd PTV D_95%_	0.046*
Conformity index	0.046*
3rd Rx V_50%_	0.260
SpinalCord D_0.03 cc_	0.260
Brainstem D_0.03 cc_	0.260
BrachPlex_R D_0.03 cc_	0.046*
BrachPlex_L D_0.03 cc_	0.260
Parotid_R D_mean_	0.260
Parotid_L D_mean_	0.181
Mandible D_0.03 cc_	0.260
Lips D_0.03 cc_	0.195
OralCavity D_mean_	0.195
Larynx D_mean_	0.344
PharynxConstr D_mean_	0.195

Abbreviations: DVH, dose‐volume histogram; PTV, planned target volume.

* Indicates significant difference (*p* < 0.05).

**TABLE A2:** Summary of tertiary PTV D_95%_ and corresponding pairwise *t*‐tests in the head and neck cohort.

D95% μ ± σ (%)					
**ASC‐0**	**ASC‐1**	**ASC‐2**	**ASC‐3**	**ASC‐4**	**ASC‐5**
80.47 ± 0.64	80.50 ± 0.63	80.51 ± 0.61	80.51 ± 0.63	80.54 ± 0.67	80.46 ± 0.64

**Pairwise *t*‐test adjusted *p*‐values**					
	**ASC‐0**	**ASC‐1**	**ASC‐2**	**ASC‐3**	**ASC‐4**
**ASC‐1**	0.233	–	–	–	–
**ASC‐2**	0.214	0.797	–	–	–
**ASC‐3**	0.214	0.764	0.887	–	–
**ASC‐4**	0.153	0.233	0.233	0.261	–
**ASC‐5**	0.807	0.250	0.191	0.191	0.031^*^

Abbreviation: ASC, aperture shape controller.

* Indicates significant difference (*p* < 0.05).

**TABLE A3:** Summary of conformity index and corresponding pairwise *t*‐tests in the head and neck cohort.

CI μ ± σ					
**ASC‐0**	**ASC‐1**	**ASC‐2**	**ASC‐3**	**ASC‐4**	**ASC‐5**
1.31 ± 0.10	1.31 ± 0.10	1.31 ± 0.09	1.31 ± 0.09	1.31 ± 0.09	1.32 ± 0.09

**Pairwise *t*‐test adjusted *p*‐values**					
	**ASC‐0**	**ASC‐1**	**ASC‐2**	**ASC‐3**	**ASC‐4**
**ASC‐1**	0.761	–	–	–	–
**ASC‐2**	0.477	0.335	–	–	–
**ASC‐3**	0.607	0.483	0.850	–	–
**ASC‐4**	0.761	0.761	0.761	0.761	–
**ASC‐5**	0.305	0.305	0.160	0.130	0.130

Abbreviation: ASC, aperture shape controller; CI, conformity index.

**TABLE A4:** Summary of BrachialPlexus_R D_0.03cc_ and corresponding pairwise *t*‐tests in the head and neck cohort.

D_0.03cc_ μ ± σ (cGy)					
**ASC‐0**	**ASC‐1**	**ASC‐2**	**ASC‐3**	**ASC‐4**	**ASC‐5**
6423 ± 432	6416 ± 428	6412 ± 432	6411 ± 428	6407 ± 429	6408 ± 426

**Pairwise *t*‐test adjusted *p*‐values**					
	**ASC‐0**	**ASC‐1**	**ASC‐2**	**ASC‐3**	**ASC‐4**
**ASC‐1**	0.336	–	–	–	–
**ASC‐2**	0.076	0.415	–	–	–
**ASC‐3**	0.074	0.415	0.806	–	–
**ASC‐4**	0.036*	0.113	0.437	0.492	–
**ASC‐5**	0.053	0.209	0.492	0.538	0.806

Abbreviation: ASC, aperture shape controller.

* Indicates significant difference (*p* < 0.05).

**TABLE A5:** Summary of post‐hoc analysis of variance results on effects of aperture shape controller settings on prostate plan dose‐volume histogram values.

DVH value	Adjusted *p*‐value
2nd PTV D_95%_	0.727
Conformity index	<0.001*
2nd Rx V_50%_	<0.001*
Bladder V_6000_	0.041*
Bladder V_5000_	0.001*
Bladder V_4000_	<0.001*
Rectum V_6000_	0.016*
Rectum V_5000_	<0.001*
Rectum V_4000_	< 0.001*
Bowel V_4000_	0.001*
Bowel D_0.03cc_	<0.001*
Femur_L D_5%_	<0.001*
Femur_R D_5%_	0.006*
PenileBulb D_0.03cc_	0.157

Abbreviations: DVH, dose‐volume histogram; PTV, planned target volume.

* Indicates significant difference (*p* < 0.05).

**TABLE A6:** Summary of conformity index and corresponding pairwise *t*‐tests in the prostate cohort.

CI μ ± σ					
**ASC‐0**	**ASC‐1**	**ASC‐2**	**ASC‐3**	**ASC‐4**	**ASC‐5**
1.16 ± 0.11	1.16 ± 0.11	1.16 ± 0.11	1.16 ± 0.11	1.16 ± 0.12	1.18 ± 0.13

**Pairwise *t*‐test adjusted *p*‐values**					
	**ASC‐0**	**ASC‐1**	**ASC‐2**	**ASC‐3**	**ASC‐4**
**ASC‐1**	0.058	–	–	–	–
**ASC‐2**	0.983	0.043*	–	–	–
**ASC‐3**	0.112	0.975	0.096	–	–
**ASC‐4**	0.093	0.644	0.093	0.584	–
**ASC‐5**	0.011*	0.028*	0.011*	0.011*	0.011*

Abbreviation: ASC, aperture shape controller; CI, conformity index.

* Indicates significant difference (*p* < 0.05).

**TABLE A7:** Summary of V_50%_ of secondary prescription corresponding pairwise *t*‐tests in the prostate cohort.

V_50%_ μ ± σ (cc)					
**ASC‐0**	**ASC‐1**	**ASC‐2**	**ASC‐3**	**ASC‐4**	**ASC‐5**
4893 ± 1106	4936 ± 1140	4913 ± 1124	4915 ± 1126	4918 ± 1151	5016 ± 1181

**Pairwise *t*‐test adjusted *p*‐values**					
	**ASC‐0**	**ASC‐1**	**ASC‐2**	**ASC‐3**	**ASC‐4**
**ASC‐1**	0.026	–	–	–	–
**ASC‐2**	0.293	0.300	–	–	–
**ASC‐3**	0.313	0.332	0.892	–	–
**ASC‐4**	0.395	0.530	0.892	0.892	–
**ASC‐5**	0.002*	0.027*	0.004*	0.004*	<0.001*

Abbreviation: ASC, aperture shape controller.

* Indicates significant difference (*p* < 0.05).

**TABLE A8:** Summary of Bladder V_6000_ and corresponding pairwise *t*‐tests in the prostate cohort.

V_6000_ μ ± σ (%)					
**ASC‐0**	**ASC‐1**	**ASC‐2**	**ASC‐3**	**ASC‐4**	**ASC‐5**
9.59 ± 6.44	9.67 ± 6.46	9.66 ± 6.50	9.64 ± 6.38	9.61 ± 6.35	9.97 ± 6.58

**Pairwise *t*‐test adjusted *p*‐values**					
	**ASC‐0**	**ASC‐1**	**ASC‐2**	**ASC‐3**	**ASC‐4**
**ASC‐1**	0.952	–	–	–	–
**ASC‐2**	0.952	0.952	–	–	–
**ASC‐3**	0.952	0.952	0.952	–	–
**ASC‐4**	0.952	0.952	0.952	0.952	–
**ASC‐5**	0.128	0.128	0.344	0.041*	0.041*

Abbreviation: ASC, aperture shape controller.

* Indicates significant difference (*p* < 0.05).

**TABLE A9:** Summary of Bladder V_5000_ and corresponding pairwise *t*‐tests in the prostate cohort.

V_5000_ μ ± σ (%)					
**ASC‐0**	**ASC‐1**	**ASC‐2**	**ASC‐3**	**ASC‐4**	**ASC‐5**
20.39 ± 10.41	20.48 ± 10.66	20.38 ± 10.58	20.61 ± 10.69	20.65 ± 10.71	21.31 ± 10.93

**Pairwise *t*‐test adjusted *p*‐values**					
	**ASC‐0**	**ASC‐1**	**ASC‐2**	**ASC‐3**	**ASC‐4**
**ASC‐1**	0.727	–	–	–	–
**ASC‐2**	0.925	0.555	–	–	–
**ASC‐3**	0.573	0.555	0.517	–	–
**ASC‐4**	0.535	0.517	0.456	0.801	–
**ASC‐5**	0.030*	0.016*	0.018*	0.016*	0.016*

Abbreviation: ASC, aperture shape controller.

* Indicates significant difference (*p* < 0.05).

**TABLE A10:** Summary of Bladder V_4000_ and corresponding pairwise *t*‐tests in the prostate cohort.

V_4000_ μ ± σ (%)					
**ASC‐0**	**ASC‐1**	**ASC‐2**	**ASC‐3**	**ASC‐4**	**ASC‐5**
35.21 ± 9.80	35.41 ± 10.04	35.22 ± 9.99	35.53 ± 10.18	35.58 ± 10.10	36.76 ± 10.15

**Pairwise *t*‐test adjusted *p*‐values**					
	**ASC‐0**	**ASC‐1**	**ASC‐2**	**ASC‐3**	**ASC‐4**
**ASC‐1**	0.446	–	–	–	–
**ASC‐2**	0.942	0.279	–	–	–
**ASC‐3**	0.426	0.548	0.279	–	–
**ASC‐4**	0.279	0.426	0.143	0.813	–
**ASC‐5**	<0.001*	<0.001*	<0.001*	<0.001*	<0.001*

Abbreviation: ASC, aperture shape controller.

* Indicates significant difference (*p* < 0.05).

**TABLE A11:** Summary of Rectum V6000 and corresponding pairwise *t*‐tests in the prostate cohort.

V_6000_ μ ± σ (%)					
**ASC‐0**	**ASC‐1**	**ASC‐2**	**ASC‐3**	**ASC‐4**	**ASC‐5**
9.94 ± 4.29	9.98 ± 4.33	9.96 ± 4.31	9.97 ± 4.25	10.02 ± 4.30	10.29 ± 4.62

**Pairwise *t*‐test adjusted *p*‐values**					
	**ASC‐0**	**ASC‐1**	**ASC‐2**	**ASC‐3**	**ASC‐4**
**ASC‐1**	0.616	–	–	–	–
**ASC‐2**	0.731	0.731	–	–	–
**ASC‐3**	0.758	0.865	0.865	–	–
**ASC‐4**	0.616	0.731	0.625	0.616	–
**ASC‐5**	0.262	0.262	0.262	0.262	0.262

Abbreviation: ASC, aperture shape controller.

**TABLE A12:** Summary of Rectum V_5000_ and corresponding pairwise *t*‐tests in the prostate cohort.

V_5000_ μ ± σ (%)					
**ASC‐0**	**ASC‐1**	**ASC‐2**	**ASC‐3**	**ASC‐4**	**ASC‐5**
17.38 ± 5.05	17.57 ± 5.25	17.44 ± 5.07	17.47 ± 5.02	17.53 ± 5.16	18.42 ± 6.24

**Pairwise *t*‐test adjusted *p*‐values**					
	**ASC‐0**	**ASC‐1**	**ASC‐2**	**ASC‐3**	**ASC‐4**
**ASC‐1**	0.421	–	–	–	–
**ASC‐2**	0.731	0.122	–	–	–
**ASC‐3**	0.796	0.731	0.861	–	–
**ASC‐4**	0.731	0.861	0.731	0.731	–
**ASC‐5**	0.060	0.060	0.057	0.057	0.057

Abbreviation: ASC, aperture shape controller.

**TABLE A13:** Summary of Rectum V_4000_ and corresponding pairwise *t*‐tests in the prostate cohort.

V_4000_ μ ± σ (%)					
**ASC‐0**	**ASC‐1**	**ASC‐2**	**ASC‐3**	**ASC‐4**	**ASC‐5**
28.39 ± 6.62	28.84 ± 7.43	28.53 ± 6.92	28.58 ± 7.13	28.85 ± 7.63	30.53 ± 9.23

**Pairwise *t*‐test adjusted *p*‐values**					
	**ASC‐0**	**ASC‐1**	**ASC‐2**	**ASC‐3**	**ASC‐4**
**ASC‐1**	0.432	–	–	–	–
**ASC‐2**	0.754	0.091	–	–	–
**ASC‐3**	0.803	0.507	0.925	–	–
**ASC‐4**	0.622	0.982	0.622	0.329	–
**ASC‐5**	0.022*	0.008*	0.006*	0.001*	0.001*

Abbreviation: ASC, aperture shape controller.

* Indicates significant difference (*p* < 0.05).

**TABLE A14:** Summary of Bowel V_4000_ and corresponding pairwise *t*‐tests in the prostate cohort.

V_4000_ μ ± σ (cc)					
**ASC‐0**	**ASC‐1**	**ASC‐2**	**ASC‐3**	**ASC‐4**	**ASC‐5**
172 ± 143	173 ± 142	171 ± 141	173 ± 142	176 ± 146	182 ± 149

**Pairwise *t*‐test adjusted *p*‐values**					
	**ASC‐0**	**ASC‐1**	**ASC‐2**	**ASC‐3**	**ASC‐4**
**ASC‐1**	0.611	–	–	–	–
**ASC‐2**	0.794	0.459	–	–	–
**ASC‐3**	0.881	0.794	0.484	–	–
**ASC‐4**	0.230	0.484	0.114	0.382	–
**ASC‐5**	0.031*	0.069	0.031*	0.069	0.043*

Abbreviation: ASC, aperture shape controller.

* Indicates significant difference (*p* < 0.05).

**TABLE A15:** Summary of Bowel D_0.03cc_ and corresponding pairwise *t*‐tests in the prostate cohort.

D_0.03cc_ μ ± σ (cGy)					
**ASC‐0**	**ASC‐1**	**ASC‐2**	**ASC‐3**	**ASC‐4**	**ASC‐5**
5150 ± 324	5149 ± 315	5133 ± 313	5153 ± 322	5164 ± 326	5222 ± 393

**Pairwise *t*‐test adjusted *p*‐values**					
	**ASC‐0**	**ASC‐1**	**ASC‐2**	**ASC‐3**	**ASC‐4**
**ASC‐1**	0.949	–	–	–	–
**ASC‐2**	0.095	0.095	–	–	–
**ASC‐3**	0.736	0.687	0.032	–	–
**ASC‐4**	0.226	0.149	0.007*	0.149	–
**ASC‐5**	0.007*	0.007*	0.004*	0.005*	0.007*

Abbreviation: ASC, aperture shape controller.

* indicates significant difference (*p* < 0.05).

**TABLE A16:** Summary of Femur_L D_5%_ and corresponding pairwise *t*‐tests in the prostate cohort.

D_5%_ μ ± σ (cGy)					
**ASC‐0**	**ASC‐1**	**ASC‐2**	**ASC‐3**	**ASC‐4**	**ASC‐5**
3431 ± 752	3433 ± 732	3426 ± 717	3474 ± 731	3486 ± 705	3590 ± 698

**Pairwise *t*‐test adjusted *p*‐values**					
	**ASC‐0**	**ASC‐1**	**ASC‐2**	**ASC‐3**	**ASC‐4**
**ASC‐1**	0.918	–	–	–	–
**ASC‐2**	0.918	0.888	–	–	–
**ASC‐3**	0.170	0.107	0.047*	–	–
**ASC‐4**	0.134	0.134	0.047*	0.816	–
**ASC‐5**	0.001*	<0.001*	<0.001*	0.001*	0.002*

Abbreviation: ASC, aperture shape controller.

* Indicates significant difference (*p* < 0.05).

**TABLE A17:** Summary of Femur_R D_5%_ and corresponding pairwise *t*‐tests in the prostate cohort.

D5% μ ± σ (cGy)					
**ASC‐0**	**ASC‐1**	**ASC‐2**	**ASC‐3**	**ASC‐4**	**ASC‐5**
3515 ± 910	3523 ± 899	3531 ± 906	3559 ± 904	3484 ± 866	3609 ± 943

**Pairwise *t*‐test adjusted *p*‐values**					
	**ASC‐0**	**ASC‐1**	**ASC‐2**	**ASC‐3**	**ASC‐4**
**ASC‐1**	0.805	–	–	–	–
**ASC‐2**	0.758	0.805	–	–	–
**ASC‐3**	0.300	0.300	0.487	–	–
**ASC‐4**	0.487	0.361	0.300	0.020*	–
**ASC‐5**	0.093*	0.062*	0.186	0.214	0.004*

Abbreviation: ASC, aperture shape controller.

* Indicates significant difference (*p* < 0.05).

**TABLE B1:** Summary of *p*‐values for MU/Dose comparisons in the head and neck cohort.

MU/Dose μ ± σ					
**ASC‐0**	**ASC‐1**	**ASC‐2**	**ASC‐3**	**ASC‐4**	**ASC‐5**
4.3 ± 1.0	4.2 ± 1.1	4.2 ± 1.0	4.2 ± 1.1	4.3 ± 1.1	4.4 ± 1.1

**Pairwise *t*‐test adjusted *p*‐values between neighboring ASC settings**					
	**ASC‐0 vs. 1**	**ASC‐1 vs. 2**	**ASC‐2 vs. 3**	**ASC‐3 vs. 4**	**ASC‐4 vs. 5**
**Both beams**	0.013*	0.415	0.019*	0.009*	0.004*

Abbreviation: ASC, aperture shape controller.

* Indicates significant difference (*p* < 0.05).

**TABLE B2:** Summary of *p*‐values for change in gantry speed comparisons in the head and neck cohort.

Pairwise *t*‐test adjusted *p*‐values between neighboring ASC settings						
	ASC‐0 vs. 1	ASC‐1 vs. 2	ASC‐2 vs. 3	ASC‐3 vs. 4	ASC‐4 vs. 5	
FF beam	0.995	0.841	0.826	0.135	0.039*	
FFF beam	0.777	0.738	0.635	0.311	0.951	

Pairwise *t*‐test adjusted *p*‐values between FF and FFF fields						
	ASC‐0	ASC‐1	ASC‐2	ASC‐3	ASC‐4	ASC‐5
FF vs. FFF	0.016*	0.019*	0.017*	0.017*	0.005*	0.003*

Abbreviations: ASC, aperture shape controller; FF, flattening filter; FFF, flattening filter‐free.

* Indicates significant difference (*p* < 0.05).

**TABLE B3:** Summary of *p*‐values for change in gantry speed comparisons in the prostate cohort.

Pairwise *t*‐test adjusted *p*‐values between neighboring ASC settings						
	**ASC‐0 vs. 1**	**ASC‐1 vs. 2**	**ASC‐2 vs. 3**	**ASC‐3 vs. 4**	**ASC‐4 vs. 5**	
**FF beam**	0.783	0.661	0.095	0.004*	0.044*	
**FFF beam**	0.552	0.742	0.316	0.165	0.919	

**Pairwise *t*‐test adjusted *p*‐values between FF and FFF fields**						
	**ASC‐0**	**ASC‐1**	**ASC‐2**	**ASC‐3**	**ASC‐4**	**ASC‐5**
**FF** ^a^ **vs. FFF**	0.001*	0.001*	0.002*	<0.001*	<0.001*	<0.001*

Abbreviations: ASC, aperture shape controller; FF, flattening filter; FFF, flattening filter‐free.

* Indicates significant difference (*p* < 0.05).

**TABLE B4:** Summary of *p*‐values for multileaf collimator speed comparisons in the head and neck cohort.

Pairwise *t*‐test adjusted *p*‐values between neighboring ASC settings						
	**ASC‐0 vs. 1**	**ASC‐1 vs. 2**	**ASC‐2 vs. 3**	**ASC‐3 vs. 4**	**ASC‐4 vs. 5**	
**FF beam**	<0.001*	0.008*	0.014*	<0.001*	0.024*	
**FFF beam**	0.001*	<0.001*	0.065	<0.001*	0.446	

**Pairwise *t*‐test adjusted *p*‐values between FF and FFF fields**						
	**ASC‐0**	**ASC‐1**	**ASC‐2**	**ASC‐3**	**ASC‐4**	**ASC‐5**
**FF** **vs. FFF**	0.118	0.303	0.514	0.196	0.148	0.009*

Abbreviations: ASC, aperture shape controller; FF, flattening filter; FFF, flattening filter‐free.

* Indicates significant difference (*p* < 0.05).

**TABLE B5:** Summary of *p*‐values for multileaf collimator speed comparisons in the prostate cohort.

Pairwise *t*‐test adjusted *p*‐values between neighboring ASC settings						
	**ASC‐0 vs. 1**	**ASC‐1 vs. 2**	**ASC‐2 vs. 3**	**ASC‐3 vs. 4**	**ASC‐4 vs. 5**	
**FF** ^a^ **beam**	<0.001^†^	0.414	<0.001^†^	<0.001^†^	<0.001^†^	
**FFF beam**	<0.001^†^	0.020^†^	0.408	0.121	0.001^†^	

**Pairwise *t*‐test adjusted *p*‐values between FF and FFF fields**						
	**ASC‐0**	**ASC‐1**	**ASC‐2**	**ASC‐3**	**ASC‐4**	**ASC‐5**
**FF** ^a^ **vs. FFF**	0.280	0.998	0.647	0.414	0.063	0.061

Abbreviations: ASC, aperture shape controller; FF, flattening filter; FFF, flattening filter‐free.

^†^ Indicates significant difference (*p* < 0.05).

**TABLE B6:** Summary of *p*‐values for edge area ratio metric comparisons in the head and neck cohort.

Pairwise *t*‐test adjusted *p*‐values between neighboring ASC settings						
	**ASC‐0 vs. 1**	**ASC‐1 vs. 2**	**ASC‐2 vs. 3**	**ASC‐3 vs. 4**	**ASC‐4 vs. 5**	
**FF beam**	<0.001*	<0.001*	<0.001*	<0.001*	<0.001*	
**FFF beam**	<0.001*	<0.001*	<0.001*	<0.001*	<0.001*	

**Pairwise *t*‐test adjusted *p*‐values between FF and FFF fields**						
	**ASC‐0**	**ASC‐1**	**ASC‐2**	**ASC‐3**	**ASC‐4**	**ASC‐5**
**FF vs. FFF**	0.108	0.771	0.923	0.457	0.109	0.887

Abbreviations: ASC, aperture shape controller; FF, flattening filter; FFF, flattening filter‐free.

* Indicates significant difference (*p* < 0.05).

**TABLE B7:** Summary of *p*‐values for edge area ratio metric comparisons in the prostate cohort.

Pairwise *t*‐test adjusted *p*‐values between neighboring ASC settings						
	**ASC‐0 vs. 1**	**ASC‐1 vs. 2**	**ASC‐2 vs. 3**	**ASC‐3 vs. 4**	**ASC‐4 vs. 5**	
**FF beam**	<0.001*	<0.001*	<0.001*	<0.001*	<0.001*	
**FFF beam**	<0.001*	<0.001*	<0.001*	<0.001*	<0.001*	

**Pairwise *t*‐test adjusted *p*‐values between FF and FFF fields**						
	**ASC‐0**	**ASC‐1**	**ASC‐2**	**ASC‐3**	**ASC‐4**	**ASC‐5**
**FF vs. FFF**	<0.001*	0.330	0.389	0.770	0.878	0.437

Abbreviations: ASC, aperture shape controller; FF, flattening filter; FFF, flattening filter‐free.

* Indicates significant difference (*p* < 0.05).

**TABLE B8:** Summary of *p*‐values for equivalent square length comparisons in the head and neck cohort.

Pairwise *t*‐test adjusted *p*‐values between neighboring ASC settings					
	**ASC‐0 vs. 1**	**ASC‐1 vs. 2**	**ASC‐2 vs. 3**	**ASC‐3 vs. 4**	**ASC‐4 vs. 5**
**Both beams**	<0.001*	<0.001*	<0.001*	<0.001*	<0.001*

Abbreviations: ASC, aperture shape controller.

* Indicates significant difference (*p* < 0.05).

**TABLE B9:** Summary of *p*‐values for equivalent square length comparisons in the prostate cohort.

**Pairwise *t*‐test adjusted *p*‐values between neighboring ASC settings**					
	**ASC‐0 vs. 1**	**ASC‐1 vs. 2**	**ASC‐2 vs. 3**	**ASC‐3 vs. 4**	**ASC‐4 vs. 5**
**Both beams**	<0.001*	<0.001*	<0.001*	<0.001*	<0.001*

Abbreviations: ASC, aperture shape controller.

* Indicates significant difference (*p* < 0.05).
